# Interaction of Bestrophin-1 and Ca^2+^ Channel β-Subunits: Identification of New Binding Domains on the Bestrophin-1 C-Terminus

**DOI:** 10.1371/journal.pone.0019364

**Published:** 2011-04-29

**Authors:** Vladimir M. Milenkovic, Sarka Krejcova, Nadine Reichhart, Andrea Wagner, Olaf Strauß

**Affiliations:** 1 Experimental Ophthalmology, Eye Hospital, University Medical Center Regensburg, Regensburg, Germany; 2 Experimentelle Ophthalmologie, Klinik und Poliklinik für Augenheilkunde, Universitätsklinikum Hamburg-Eppendorf, Hamburg, Germany; University of Queensland, Australia

## Abstract

Bestrophin-1 modulates currents through voltage-dependent L-type Ca^2+^ channels by physically interacting with the β-subunits of Ca^2+^ channels. The main function of β-subunits is to regulate the number of pore-forming Ca_V_-subunits in the cell membrane and modulate Ca^2+^ channel currents. To understand the influence of full-length bestrophin-1 on β-subunit function, we studied binding and localization of bestrophin-1 and Ca^2+^ channel subunits, together with modulation of Ca_V_1.3 Ca^2+^ channels currents. In heterologeous expression, bestrophin-1 showed co-immunoprecipitation with either, β3-, or β4-subunits. We identified a new highly conserved cluster of proline-rich motifs on the bestrophin-1 C-terminus between amino acid position 468 and 486, which enables possible binding to SH3-domains of β-subunits. A bestrophin-1 that lacks these proline-rich motifs (ΔCT-PxxP bestrophin-1) showed reduced efficiency to co-immunoprecipitate with β3 and β4-subunits. In the presence of ΔCT-PxxP bestrophin-1, β4-subunits and Ca_V_1.3 subunits partly lost membrane localization. Currents from Ca_V_1.3 subunits were modified in the presence of β4-subunit and wild-type bestrophin-1: accelerated time-dependent activation and reduced current density. With ΔCTPxxP bestrophin-1, currents showed the same time-dependent activation as with wild-type bestrophin-1, but the current density was further reduced due to decreased number of Ca^2+^ channels proteins in the cell membrane. In summary, we described new proline-rich motifs on bestrophin-1 C-terminus, which help to maintain the ability of β-subunits to regulate surface expression of pore-forming Ca_V_ Ca^2+^-channel subunits.

## Introduction

Bestrophin-1 is an anion channel [1] which can also regulate voltage-dependent Ca^2+^ channels [2], [3], [4], [5]. The regulatory effects include modulation of activation kinetics [2], [3], [5], voltage-dependent activation [3] and/or current amplitude [4], [5].

Voltage-dependent Ca^2+^ channels are composed of pore-forming Ca_V_-subunits (α1-subunits) which determine the basic Ca^2+^ properties and of the auxiliary β, α2δ- and sometimes the γ-subunits [6], [7]. The Ca^2+^ channel β-subunits have complex functions [8], [9]: they modulate the electrophysiological properties of the pore-forming Ca_V_-subunits, interact with kinases and are required for the transport of Ca_V_-subunits to the cell membrane. It is most likely that described effects of bestrophin-1 on L-type channel activity are due to modulation of β-subunit function. Using heterologous co-expression of L-type Ca^2+^ channels and C-terminus fragments of bestrophin-1, proline-rich motifs between the amino acid positions 330 and 346 were identified to enable interaction with β-subunits of voltage-dependent Ca^2+^ channels via SH3 domains [4], [5] . The physical interaction of full-length bestrophin-1 with Ca^2+^ channel β-subunits was confirmed by Reichhart et al. [5].

Mutations in the gene for bestrophin-1, BEST1, lead to different types of retinal or macular degenerations [1], [10]. The most common phenotype is Best’s vitelliforme macular dystrophy [11], [12], [13]. Symptoms include macular degeneration, fast accumulation of lipofuscin and a reduction of the so called “light-peak” in the patient’s electro-oculogram [14]. However, little is known about how mutations in BEST1 lead to disease. It is possible that BEST1’s role as an anion channel and/or Ca^2+^ channel regulator could explain the typical changes seen in the electro-oculogram. Several lines of evidence show that the light-peak in the electro-oculogram is generated by activation of Cl^−^ conductance and is dependent on the presence of β4- and Ca_V_1.3 subunits [3], [15], [16], [17] in the retinal pigment epithelium (RPE) which closely interacts with the photoreceptors in the retina [18]. Bestrophin-1 is known to be basolaterally located in the RPE and can function as both Cl^−^ channel and Ca^2+^ channel regulator. The combined function as a Ca^2+^-dependent anion channel and Ca^2+^ channel regulator would provide an efficient feedback loop to control Ca^2+^-dependent Cl^−^ transport, for example, by the RPE [1], [18]. There are several studies which investigated eyes from Best patients [19], [20], [21], [22], [23], [24]. So far, only one pathologic effect of a mutant bestrophin-1 has been found. This mutant form does not show uniform basolateral localization [20]. Thus, the proper trafficking of bestrophin-1 and probably its interaction partners seem to be important for understanding bestrophin-1 function in disease. In this regard, determining the influence of bestrophin-1 on β-subunits derived regulation of pore-forming Ca_V_1.3-subunits in the plasma membrane would allow for better understanding of Best’s disease.

The aim of our study is to first investigate the interaction of full-length bestrophin-1 with β-subunits and secondly, the influence of bestrophin-1 on the ability of β-subunits to regulate the surface expression of Ca_V_-subunits. In order to test this hypothesis we performed immunoprecipitation experiments with heterologously expressed bestrophin-1, β-subunits and α1-subunit Ca_V_1.3 corresponding to the Ca^2+^ channel expressed in the RPE [3], [25]. These interactions and the influence on membrane localization of Ca_V_1.3-subunits were verified by correlation with the subcelluar localization using confocal microscopy. The functional effects were studied by patch-clamp analysis of Ca^2+^ channel currents from heterologously expressed Ca_V_1.3-subunits and β4-subunits.

## Materials and Methods

### Cell culture and transfection

CHO (ATCC, cat# CCL-61) cells were cultured in 100-mm culture dishes in Hams F12 medium (Invitrogen). HEK-293 (ATCC, cat# CRL-1573), and COS-7 cells (ATCC, cat# CRL-1651) were cultured in Dulbecco’s modified Eagle’s medium (D-MEM) containing L-glutamine, 4500 mg/l glucose and 110 mg/l sodium pyruvate. ARPE-19 (ATCC, cat# CRL-2302) cells were cultured in DMEM/Ham's F-12 medium, 50∶50 mixture supplemented with insulin/transferin, non essential amino acids, and 15 mM HEPES buffer (Invitrogen). All media were supplemented with 10% (v/v) fetal calf serum (Invitrogen), and 1% (v/v) penicillin-streptomycin (Invitrogen). Cells were cultured at 37°C, with relative humidity of 95% and 5% CO_2_ concentration. Transfections were carried out using Lipofectamine 2000 transfection reagent (Invitrogen) following the manufacturer's instructions or by microinjections. Microinjections were performed using a FemtoJet Injector in conjunction with a InjectMan Manipulator (both Eppendorf, Hamburg, Germany). The detailed description is included in the chapter “Ca_V_1.3 patch-clamp recordings”.

### Plasmid Constructs

1. Human bestrophin-1, BEST1 [Homo sapiens; NM_004183], hBest-pEGFP-N1 (N-terminal EGFP-tagged human bestrophin), hBest-pcDNA3.1 2. calcium channel, voltage-dependent, β1 (Rattus norvegicus NM_017346), β3 (Rattus norvegicus; NM_012828), and β4-subunit (Rattus norvegicus; Gene ID 25297 and 58942); depending on experimental conditions tagged either with His or c-Myc), β3-pCMV, and β4-pCDNA3; 3. Cacna1d, calcium channel, voltage-dependent, L-type, α1D subunit Ca_V_1.3 (Homo sapiens: NM_000720.2) α1D subunit CaV1.3-GFP; 4. α2δ1-pcDNA3 (Gene ID 776), 5. eGFP pcDNA3 reporter plasmid was used as transduction control. Ca^2+^ channel constructs were provided by Prof. Striessnig (Innsbruck). Human bestrophin-1 constructs were provided by Prof. Weber (Regensburg). All described constructs were sequenced for integrity, and pure plasmid DNA was isolated by using a plasmid maxi kit (Qiagen).

### DNA manipulations

Deletion of the C-terminal proline rich (CT-PxxP) region between 462-575aa (113 amino acids) was introduced into human bestrophin-1 by PCR using the following primers: 5′- ATCGCTCGAGCCACCATGACCATCACTTACACA and 5′- CGATGGATCCATGGCAGACTTGAAGGCGTC. Resulting PCR product was digested using XhoI and BamHI restriction enzymes, and subsequently inserted into pCDNA3-bestrophin-1 plasmid digested with XhoI/BamHI. All the constructs were verified by DNA sequencing.

### Antibodies

Proteins were detected using the following antibodies: mouse monoclonal anti-human-bestrophin ab2182, mouse monoclonal anti His6 ab18184, rabbit polyclonal anti His6ab9108 (Abcam plc, Cambridge, UK), rabbit anti-β3, rabbit Cav1.3 (Alomone Labs), goat anti Cav1.3 (Santa Cruz), mouse monoclonal anti beta-actin (clone JLA20, Hybridoma Bank Iowa), and mouse monoclonal anti-GFP (Roche). Rabbit polyclonal anti bestrophin-1 antibody was provided by Prof. Dr. Karl Kunzelmann (Regensburg). Proteins were visualized by Western blot using HRP-conjugated goat anti-rabbit or goat anti-mouse antibodies (New England Biolabs).

### Immunoprecipitation

Subconfluent (70-80%) culture of CHO, HEK-293 or COS-7 cells were transiently transfected with combinations of plasmids encoding a human-bestrophin-1 (N-terminal EGFP-tagged or untagged), β3 (untagged), β4-His_6_ and Ca_V_1.3 (N-terminal eGFP-tagged or untagged) subunits of voltage-dependent calcium channels using Lipofectamine 2000 transfection reagent (Invitrogen) according to the manufacturer's protocol. The pcDNA3.1 and pEGFP plasmids were used as negative control. After a 24-h incubation, cells were washed with 1xPBS and then lysed in culture dish with shaking for 15 min at 4°C with ice-cold lysis buffer (150 mM Tris-HCl, pH 7.5, 150 mM NaCl, 1% Nonidet-P40, 0.5% natrium deoxycholate, 1 tablet Complete Mini protein inhibitor mixture/10 ml (Roche Applied Science), and 0.7 µg/ml pepstatin). Cell lysate was scraped and transferred to a new tube and lysed for additional 15 min at 4°C with rocking. The lysates were clarified by centrifugation at 13,000× *g* for 10 min at 4°C.

### Pre-clearing

Supernatants were applied to 50 µl of Protein G-Agarose (Sigma) and incubated 3 hours with rocking at 4°C. After pre-clearing and centrifugation at 13,000× g for 1 min the lysates were applied to new tubes with 50 µl of Protein G-Agarose already incubated for 1 hour with 3 µg of suitable/relevant antibody. Subsequently, after overnight incubation on a rotating wheel at 4°C the beads were washed three times with washing buffer. All centrifugation steps were carried out at 1,200× g for 1 min at 4°C. 1. Washing buffer (50 mM Tris-HCl, pH 7.5, 150 mM NaCl, 1% Nonidet-P40, 0.5% natrium deoxycholate, 1 tablet Complete Mini protein inhibitor mixture/10 ml (Roche Applied Science) and 0.7 µg/ml pepstatin). 2. Washing buffer (50 mM Tris-HCl, pH 7.5, 250 mM NaCl, 0.1% Nonidet-P40, 0.05% natrium deoxycholate). 3. Washing buffer (50 mM Tris-HCl, pH 7.5, 50 mM NaCl, 0.1% Nonidet-P40, 0.05% natrium deoxycholate). Protein complexes were dissociated from beads by incubation at 37°C for 30 min in 4xSDS loading buffer. The immunoprecipitates were subjected together with total lysates to 7.5% or 10% SDS-PAGE and Western blot was carried out.

### Western Blot Analysis

Western Blot analysis were performed as previously described in detail [3]. Lysates of membrane proteins were prepared by three freezing and thawing steps (liquid N_2_; 42°) and two centrifugation steps at 500 and 43.000 g, the pellet was suspended in lysis buffer and subjected to SDS-PAGE (7.5–10% gel) The proteins were blotted to nitrocellulose filter membranes (Polyscreen; NEN Life Science Products, Boston, MA). The blots were blocked in 5% non-fat dry milk and 5% bovine serum albumin. Primary antibodies were diluted as follow: anti-human-bestrophin, anti-β3, and anti-GFP (1∶5000), anti His6 (1∶2500), anti Cav1.3, and anti beta-actin (1∶1000). After incubation with primary antibodies, blots were visualized with a peroxidase-conjugated secondary antibody and a chemiluminiscence kit according to 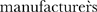 instructions. Chemi-luminescence detection (*of bound secondary antibodies)* was carried out using Immobilon Western HRP substrate detection kit (Millipore). The images were digitalized using an image analyzer (Chemimager, Biozym).

### Immunohistochemistry and confocal microscopy

For immunofluorescence experiments, transiently transfected CHO and ARPE-19 cells were grown on a sterile glass cover slip. 24 hours after the transfection cells were washed with 1× PBS, and fixed for 10 min at room temperature with 4% (w/v) para-formaldehyde. After three additional washing steps with 1× PBS, cells were permeabilized with blocking/permeabilization solution [10% (v/v) normal goat serum, 0.5% (v/v) Triton X-100 in 1× PBS) for 30 min. Cells were then labeled for 1 hour with anti-bestrophin-1 antibody, and anti-β3 antibody diluted 1∶500, and goat anti-Ca_v_1.3 antibody diluted 1∶100 in 2% normal goat serum, 0.1% Triton X-100 in 1× PBS. After three additional washing steps cells were incubated for 1 hour with appropriate secondary antibodies diluted 1∶500 (conjugated with Alexa 488, Alexa 546, and Alexa 633, (Invitrogen). Cells were mounted in confocal matrix (Micro Tech Lab, Graz, Austria) and then examined using confocal microscope LSM510 (Carl Zeiss, Göttingen, Germany).

### Quantitative c-olocalization analysis

For the quantitative co-localization analysis, ARPE-19 cells grown on glass cover slips were either double or triple transfected with various bestrophin constructs and labeled with corresponding primary antibody. After subsequent incubation with secondary antibodies conjugated with Alexa 488, 546, and 633 diluted 1∶500 (Invitrogen, Germany), cover slips were examined using confocal microscope LSM 510 (Zeiss, Göttingen, Germany). Confocal microscopy has advantage over the standard fluorescence microscopy, because it generates thin optical sections and thus allows quantification of the co-localization of antigens. Triple fluorescence for green, red and infrared channels was obtained using excitation of an argon-helium-neon laser at wave lengths of 488, 546, and 633 nm. Emission of the different fluorophores was detected using appropriate filter sets and multi channel acquisition. Triple stained images were obtained by sequential scanning for each channel to eliminate the crosstalk of chromophores and to ensure reliable quantification of co-localization. Images were recorded at intensity levels below saturation, estimated by intensity analysis module. Confocal images were quantitatively analyzed using an ImageJ software package. Pearson's correlation coefficient (PCC) was employed to evaluate co-localization according to Abramoff [26]. PCC is one of the standard techniques applied in pattern recognition for matching one image to another in order to describe the correlation of the intensity distributions between channels. It takes into consideration only for the similarity of shapes between two images, and does not depend upon image pixel intensity values. Values of PCC are defined from -1 to 1 where -1 indicates no overlap and 1 is a complete co-localization. For surface expression analysis, confocal image files were loaded into ImageJ (version.1.45b), and were submitted to edge detection process using built in edge detection algorithm (3×3 Sobel edge filter). In the next step, singe cells were selected and cell surface was labelled using freehand tool. Intracellular regions were additionally selected, and all selected regions were saved as a region of the interest (ROI). Total number of pixels were counted using analyze particles command for each channel separately. The number of pixels from the whole cell was subtracted from the intracellular regions, thus giving the proportion of the pixel localized to the membrane. Furthermore, membrane pixel values were divided with intracellular pixel values, giving relative surface expression.

### Patch-Clamp recordings of Ca_V_1.3 channel currents - Microinjection

For patch-clamp experiments and confocal microscopy CHO cells were microinjected with plasmids coding Ca^2+^ channel subunits and different bestrophin-1 depending on the experiment as indicated in the results. Microinjections were performed under an inverted microscope (Carl Zeiss) equipped with a micromanipulator InjectMan NI2 (Eppendorf) by using an automated FemtoJet (Eppendorf) and Femtotip (Eppendorf) glass microcapillaries. CHO cells were plated on 12-mm glass plates and microinjected with plasmid DNA (50 ng/µl for each construct). Immediately after microinjection, cells were incubated overnight at 30°C, and next day cells were transferred into 37°C cell culture incubator. *Patch-clamp analysis:* Membrane currents were measured in the whole-cell configuration of the patch-clamp technique. While recording, transfected cells were superfused in a bath solution containing (mM): choline chloride 150, BaCl_2_ 10, MgCl_2_ 1, HEPES 10; pH 7.4 adjusted with CsOH; 333mOsm). To elicite voltage-dependent currents, cells were stimulated from a holding potential of −70 mV by stepwise depolarization. Gating currents were measured according to Fan et al. [27]. To measure gating currents, the Ca_V_1.3 pore was blocked using 10 mM Co^2+^, which was used to replace Ba^2+^. Co^2+^ is known to block L-type channels as it is not able to permeate through the pore [27]. Before measuring the gating current amplitude the Ca_V_1.3 currents were measured currents using Ba^2+^ as charge carrier. Only cells which showed a strong and robust Ba^2+^ current were used for gating current analysis. For proper measurement of gating currents, special care was taken to compensate for residual capacitative currents. The perfusion chamber was assessed by fluorescent microscope. Transfected cells were selected by their GFP fluorescence. For whole-cell recording, patch-pipettes of 3–5 MΩ were made from borosilicate tubes using a DMZ-Universal Puller (Zeitz, Augsburg Germany). Pipettes were filled with a pipette-solution containing (mM): CsCl 135, MgCl_2_ 1, CsEGTA 10; pH 7.4 adjusted with CsOH; 283 mOsm). Membrane currents were recorded using an EPC-10 computer-controlled patch-clamp amplifier in conjunction with the TIDA software for data acquisition and analysis. The mean membrane capacitance was 22.04±1.3 pF (n = 25). The access resistance was compensated for to values lower than 10 MΩ. Analysis of voltage-dependent activation was done by plotting steady-state currents against membrane potentials of electrical stimulation. Individual cell plots were fitted using the Boltzmann equation. Gating current amplitudes were plotted against the voltages of the electrical stimulation. For comparison gating current density was calculated at +20 mV.

### Calculations and statistical analysis

Experiments were repeated at least three times. The Western blots shown in the figures show a representative experiment. Mean values were given as mean +/− SEM; n refers to the number of experiments. Statistical difference was tested by ANOVA; statistic significant difference was considered at p values smaller than 0.05.

## Results

**Figure 1:**
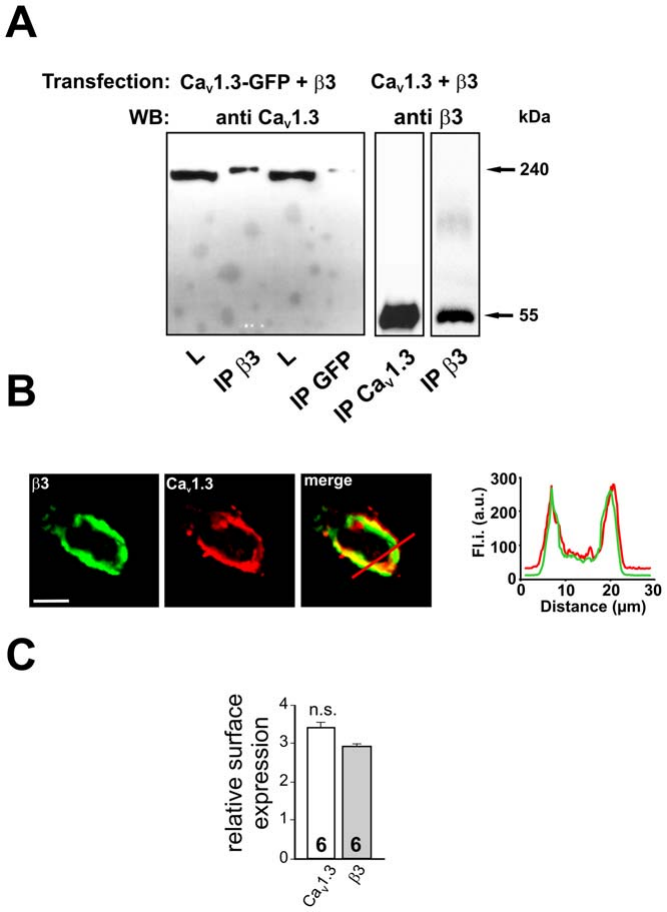
Interaction between pore-forming Ca_V_1.3 and auxiliary β-subunits of voltage-dependent Ca^2+^ channels. **1A: Left panel**: CHO cells were transfected with Ca_V_1.3-GFP fusion construct and β3-subunits, precipitated with antibodies against β3-subunits and blotted for Ca_V_1.3 protein. Proteins precipitated using antibodies against GFP were positively stained with antibodies against Ca_V_1.3 indicating identification of Ca_V_1.3 subunits. **Right two panels**: CHO cells transfected with Ca_V_1.3 and β3-subunits; Ca_V_1.3 was immunoprecipitated and blotted for β3-subunits. Control experiment: precipitation using anti-β3-antibody and blot stained against β3-subunit. (L  =  lysate, 10% of total protein; IP  =  immunoprecipitation) **1B**: ARPE-19 cells transfected with β3-subunit (green) and Ca_V_1.3 (red). The merged picture shows co-localization of β3-subunits and Ca_V_1.3. On the right: fluorescence profile showing subcellular protein distribution. **1C**: To quantify plasma membrane localization, pixel analysis was performed for edge detection to calculate surface expression (data are mean ± SEM; n = 3). Scale bar represents 10 µm.

In order to determine the possible physical interaction between Ca^2+^ channel subunits and bestrophin-1, immunoprecipitation experiments were performed using heterologously expressed proteins. All proteins were determined to have no endogeneous expression in CHO cells (Figure S1A). We first tested our experimental settings using known physiological interaction between pore-forming Ca_V_1.3 subunits and accessory β-subunits [6], [7] (Figure 1). CHO cells were co-transfected with Ca_V_1.3 subunits and β3-subunits. Immunoprecipitation of β3-subunits showed the presence of Ca_V_1.3 subunits (Figure 1A, left panel) and vice versa (Figure 1A, right panels). Using immunocytochemistry, we studied possible interaction of these proteins in intact cells (Figure 1B). ARPE-19 cells were chosen for co-localization experiments because bestrophin-1 is normally expressed in the RPE and protein trafficking differs in the RPE of other cell types [18]. ARPE-19 cells were transfected with Ca_V_1.3 subunits and β3-subunits and their subcellular localization was investigated by confocal microscopy. Both Ca_V_1.3 and β3-subunits were found in the cell membrane. To quantify the co-staining and co-localization of the two proteins in the cell membrane, Pearson’s correlation coefficient was calculated. The resulting coefficient of 85.0±8% (n = 3; Table 1) indicates a good co-localization. In order to further analyze membrane localization, we analyzed fluorescence profiles across the cells whilst avoiding the nuclear region. The confocal images were used to quantify the plasmalemmal localization by pixel analysis using edge detection (Figure S2A–C). This revealed a relative surface expression of 3.4±0.13 for Ca_V_1.3 and 2.9±0.6 for β3-subunits (n = 3; n.s.) (Figure 1C).

**Table 1:** Summary of Pearson’s correlation coefficients to show co-localization of Ca^2+^ channel subunits and bestrophin-1 in confocal pictures.

Transfection	Pearsonss co-localization coefficient*	Cells
Cav1.3 β3	85±8	ARPE-19
β3 +Best1	64±7	ARPE-19
Cav1.3+β3+Best1	63.5±5	ARPE-19
Cav1.3+β4-His+Best1	59±6	ARPE-19
Cav1.3+β4-His+Best1	57±4	CHO
Cav1.3+β4-His+ΔCTPxxP-Best1	42±5	CHO
P2Y2-His+ΔCTPxxP-Best1	14±4	CHO

**Figure 2:**
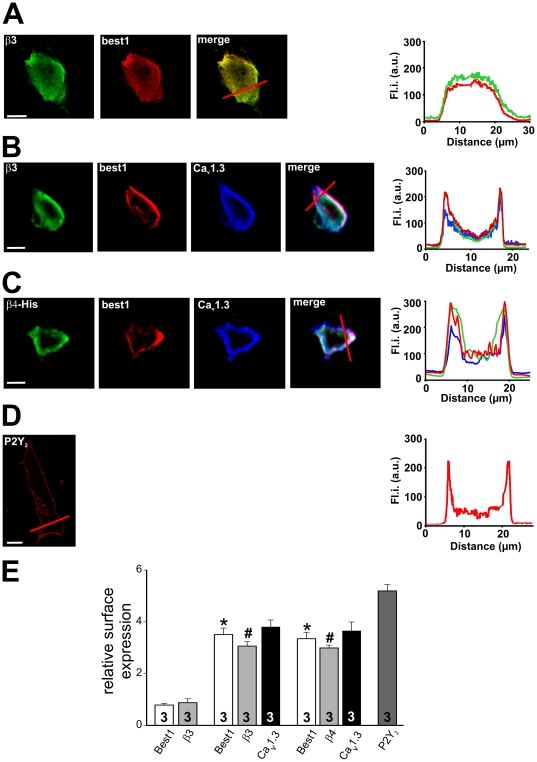
Subcellular localization of heterologously expressed Ca_V_1.3, β-subunits of voltage-dependent Ca^2+^ channels and bestrophin-1: ARPE-19 cells were transfected with: β3-subunits and bestrophin-1, Ca_V_1.3, β3 or β4-subunits and bestrophin-1, and human P2Y_2_-His_6_ receptor. **2A**: Cells transfected with β3-subunit (green) and bestrophin-1 (red). Yellow colour in the merged picture indicates interaction of both proteins. On the right: fluorescence profiles showing subcellular protein distribution. **2B**: Cells transfected with β3-subunit (green), bestrophin-1 (red), and Ca_v_1.3 subunit (blue). White colour in the merged picture suggests co-localization of all three proteins. On the right: fluorescence profiles showing subcellular protein distribution. **2C**: Cells transfected with β4-subunit (green), bestrophin-1 (red), and Ca_v_1.3 subunit (blue). White colour in the merged picture suggests co-localization of all three proteins. On the right: fluorescence profiles showing subcellular protein distribution. **2D**: Human P2Y_2_-His_6_ receptor which shows plasma membrane localization as a control. Note: cells which express Ca_V_1.3 and β-subunits always appear in a more spherical shape and do not remain flat due to the expression of the large L-type channel subunits. The smaller P2Y_2_-receptor did not change the cell shape. **2E**: Relative surface expression quantified by edge detection analysis (data are mean ± SEM; n = 3). (*  =  p<0.05 for bestrophin-1; #  =  p<0.05 for β3-subunits, unpaired t-test) Scale bar represents 10 µm.

In order to detect the interaction of bestrophin-1 and β-subunits, co-localization studies were performed in intact cells (Figure 2) in the same way as described for interaction of Ca_V_1.3 and β-subunits. ARPE-19 cells were co-transfected with β3-subunits and bestrophin-1. In these cells, the majority of the two proteins showed a good co-localization (Table 1) and was diffuse across the cytosol (Figure 2A). Measurement of the relative surface expression by pixel analysis revealed values of 0.78±0.06 for β3-subunits and 0.877±0.15 for bestrophin-1 (Figure 2E; n = 3; n.s.) indicating a cytoplasmic localization for β3-subunits and bestrophin-1 when expressed together. When ARPE-19 cells were co-transfected with bestrophin-1, β3-subunits and Ca_V_1.3 subunits, all three proteins were located in the cell membrane (Figure 2B, Table 1). This differs significantly compared to the bestrophin-1 and β3-subunit localization shown before. The localization of the three proteins is shown through pixel analysis which revealed a good surface expression with values of 3.80±0.26 for Ca_V_1.3, 3.06±0.16 for β3-subunits and 3.51±0.25 for bestrophin-1 (all n = 3; Figure 2E). The same could be observed in cells co-transfected with Ca_V_1.3, β4-subunits and bestrophin-1 (Figure 2C). The relative surface expression were 3.65±0.34 for Ca_V_1.3, 2.99±0.10 for β4-subunits and 3.34±0.25 for bestrophin-1 (all n = 3; Figure 2E). For comparison and validation of the fluorescence ratios, the ratio for the purinergic receptor P2Y_2_-His_6_, a typical membrane protein, was calculated (Figure 2D). This protein showed a relative surface expression of 5.19±0.24 (Figure 2E).

**Figure 3:**
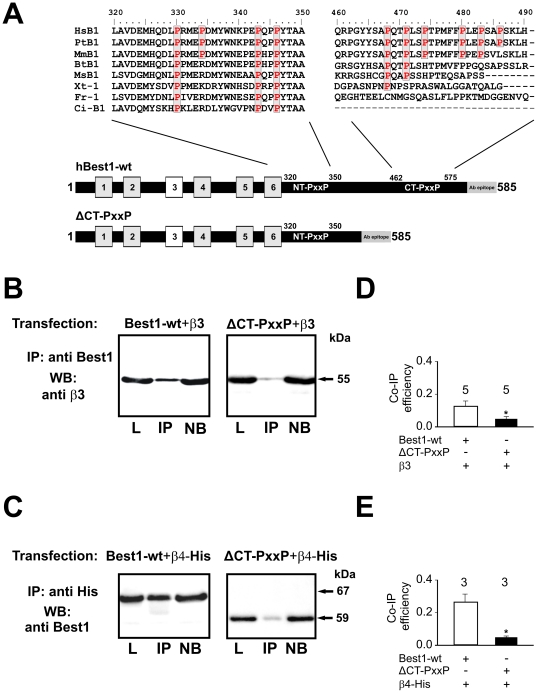
Detection of interaction sites between β-subunits and bestrophin-1. **3A**: Bestrophin-1 construct used in this study and alignment of amino acid sequences of the C-terminus of the bestrophin-1 from different species (Boxes: transmembrane domains). Two among vertebrate species highly conserved clusters of proline-rich motifs (PxxP) could be detected. In the ΔCTPxxP mutant form, PxxP motifs between amino acid 468 to 486 were removed with unchanged recognition sites for the anti bestrophin-1 antibody. **3B**: HEK-293 cells were transfected with β3-subunits together with bestrophin-1 or ΔCTPxxP constructs. Proteins were precipitated using anti-bestrophin-1 antibody and blots were visualized for anti-β3-subunit to show co-immunoprecipitation. **3C**: HEK-293 cells were transfected with His-tagged β4-subunits together with bestrophin-1 wild type or ΔCTPxxP constructs. Proteins were precipitated using anti-His antibody and the blots were visualized with anti-bestrophin-1 antibody to show co-immunoprecipitation. **3D**: Relative co-immunoprecipitation of β3-subunits with either wild-type or ΔCTPxxP bestrophin-1: efficiency was measured by densitometry (n = 5). **3E**: Relative co-immunoprecipitation of β4-subunits with either wild-type or ΔCTPxxP bestrophin-1 (depicted n = 3). (L  =  lysate; IP  =  immunoprecipitation; NB  =  not bound). The following species abbreviations were used: Hs, Homo sapiens, Mm, Macaca mulatta, Bt, Bos taurus, Ms, Mus musculus, Xt, Xenopus tropicalis, Fr, Fugu rubripes, and Ci, Ciona intestinalis.

To identify the mechanism of interaction between bestrophin-1 and β-subunits of Ca^2+^ channels, bestrophin-1 sequences were analyzed for interaction domains (Figure 3A). We searched for proline-rich (PxxP) motifs which could bind to the SH3-domain of the β-subunits. Together with the already known cluster of PxxP motifs between amino acid position 330 and 346 on bestrophin-1 C-terminus [4] we detected a cluster of 4 proline-rich motifs, which are highly conserved among many species, between the amino acid positions 468 and 486. To explore the role of the newly detected cluster, we generated a deletion mutant of bestropin-1 lacking the proline-rich motifs between amino acid positions 462 and 575 (named ΔCT-PxxP). Using this mutant, immunoprecipitation experiments were performed to analyze binding between several β-subunits and mutant bestrophin-1. For this purpose, HEK-293 cells were transfected with wild-type or with mutant bestrophin-1 together with β3- or β4-subunits. Wild-type bestrophin-1 could be co-immmunoprecipitated with either β3- or β4-subunits (Figures 3B and 3C). Similar results were obtained using CHO or COS-7 (Figure S1B). Western blot analysis of the precipitates using antibodies directed against bestrophin-1 showed that ΔCT-PxxP could be precipitated with the same efficiency as the wild-type bestrophin-1 (Figure S3A). These precipitates were further analyzed for the presence of either β3-subunits or β4-subunits showing co-precipitation. The ΔCT-PxxP bestrophin-1 also showed co-precipitation with β3-subunits or β4-subunits. Based on the summarized amount of protein in the lysate, precipitation fraction and non-bound fraction, we calculated the relative co-precipitation efficiency of the ΔCT-PxxP mutant and β3-subunits (Figures 3D and 3E). β3-subunit showed a significant decrease in co-precipitation efficiency from 12±3% with wild-type bestrophin-1 to 4±1% (n = 5; p = 0.035 unpaired t-test) with the ΔCT-PxxP mutant of bestrophin-1. The β4-subunits also showed a significant decrease in co-precipitation efficiency from 26.6±6% with wild-type bestrophin-1 to 4±0.1% with ΔCTPxxP bestrophin-1 (n = 3; p = 0.02 unpaired t-test, Figure S3B).

**Figure 4:**
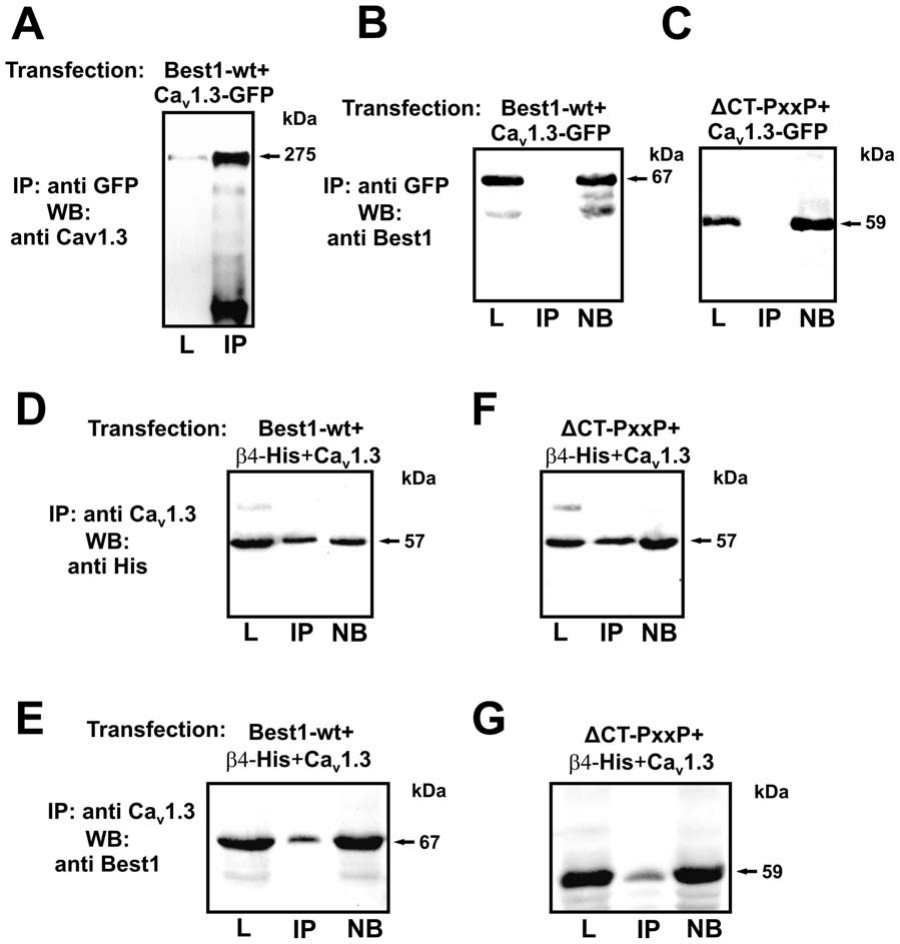
Complex formation of Ca_V_1.3, β4-subunits and bestrophin-1. HEK cells were transfected with Ca_V_1.3, His-tagged β4-subunits, bestrophin-1 or ΔCTPxxP bestrophin-1. Immunoprecipitation was performed using anti-Ca_V_1.3 antibodies, precipitates were analyzed by Western blot. **4A**: Transfection: Ca_V_1.3 and bestrophin-1. Blot staining with anti-Ca_V_1.3 antibody to show efficient precipitation of Ca_V_1.3 subunits. **4B**: Transfection: Ca_V_1.3 and bestrophin-1. Blot staining with anti-bestrophin-1 antibody showing no co-precipitation of Ca_V_1.3 subunits with bestrophin-1. **4C**: Transfection: Ca_V_1.3 and ΔCTPxxP bestrophin-1. Blot staining with anti-bestrophin-1 antibody showing no co-precipitation of Ca_V_1.3 subunits with ΔCTPxxP bestrophin-1. **4D**: Transfection: Ca_V_1.3, His-tagged β4-subunits and bestrophin-1. Blot staining with anti-His antibody showing co-precipitation of Ca_V_1.3 subunits with β4-subunits. **4E**:Transfection: Ca_V_1.3, His-tagged β4-subunits and bestrophin-1. Blot staining with anti-bestrophin-1 antibody showing indirect co-precipitation of Ca_V_1.3 subunits with bestrophin-1. **4F**: Transfection: Ca_V_1.3, His-tagged β4-subunits and ΔCTPxxP bestrophin-1. Blot staining with anti-His antibody showing co-precipitation of Ca_V_1.3 subunits with β4-subunits. **4G**: Transfection: Ca_V_1.3, His-tagged β4-subunits and ΔCTPxxP bestrophin-1. Blot staining with anti-bestrophin-1 showing indirect co-precipitation of Ca_V_1.3 subunits with ΔCTPxxP bestrophin-1. (L  =  lysate; IP  =  immunoprecipitation; NB  =  not bound)

As shown by immunohistochemistry, Ca_V_1.3, β-subunit and bestrophin-1 possibly form complexes. Thus, we analyzed this further by immunoprecipitation. HEK cells were double transfected by Ca_V_1.3 together with bestrophin-1 or the ΔCTPxxP mutant form. Ca_V_1.3 subunits were precipitated (Figure 4A) and analyzed by Western blot for the presence of either bestrophin-1 or ΔCTPxxP bestrophin-1 (Figure 4B/C). The Western blots revealed no co-precipitation with Ca_V_1.3 subunits. In contrast, indirect co-precipitation of either bestrophin-1 or ΔCTPxxP bestrophin-1 with Ca_V_1.3 subunits was observed in the presence of β4-subunits. HEK cells were triple transfected with Ca_V_1.3, β4-subunits and with either bestrophin-1 or ΔCTPxxP bestrophin-1. Ca_V_1.3 subunits were precipitated and analyzed by Western blot. In this case, Western blot analysis revealed co-precipitation of Ca_V_1.3 subunits with β4-subunits and bestrophin-1 (Figure 4 D/E). In addition, also co-precipitation of Ca_V_1.3 subunits with β4-subunits and ΔCTPxxP bestrophin-1 was observed (Figure 4 F/G).

**Figure 5:**
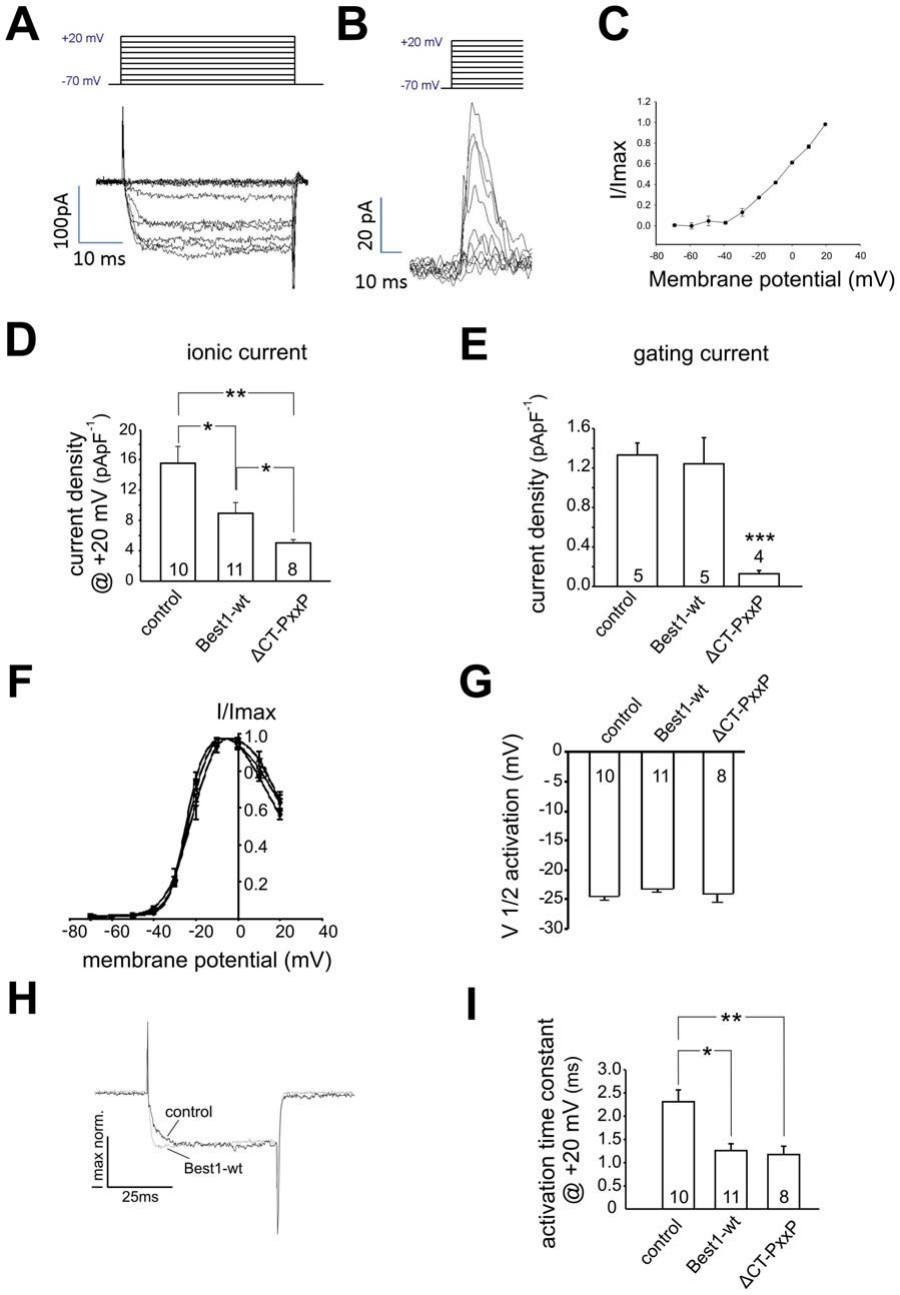
Patch-Clamp analysis of Ca_V_1.3/β4 currents under influence of bestrophin-1. **5A**: Whole cell Ba^2+^-currents (lower panel) measured from CHO cells expressing Ca_V_1.3, β4-, α2δ1-subunits. Currents were elicited by a series of 9 voltage-steps of 10 mV increasing amplitude and 50 ms duration from a holding potential of -70 mV (upper panel). **5B**: Gating currents: Currents elicited by the electrical stimulation as shown in 5A in the same cell after switching to Co^2+^ containing bath solution. **5C**: Plot of normalized gating currents to the membrane voltages of the electrical stimulation (mean ± SEM, n = 3). **5D**: Comparison of the ionic current density of L-type currents from heterologously expressed Ca^2+^ channel proteins and different bestrophins measured at +20 mV (note: the values are close to those we have published [5] but represent a different set of data). **5E**: Comparison of the gating current density in the presence of 10 mM Co^2+^ from heterologously expressed Ca^2+^ channel proteins and different bestrophins measured at +20 mV. **5F**: Normalized current/voltage plots of L-Type currents either in the absence, presence of bestrophin-1 or the ΔCTPxxP mutant bestrophin-1, fit by Boltzmann equation. **5G**: Comparison of the voltages of half maximal activation obtained from Boltzmann fits of the curves in Fig. 5F. **5H**: Whole cell Ba^2+^-currents measured from CHO cells expressing either Ca_V_1.3, β4-, α2δ1 subunits or Ca_V_1.3, β4-, α2δ1 subunits plus wt-bestrophin-1. Currents were elicited by a voltage-jump from -70 mV to +20 mV. The recording shows the currents normalized to their maximal amplitude for comparison. **5I**: Comparison of the time-dependent activation of L-type currents from different heterologously expressed Ca^2+^ channel proteins and bestrophins measured as time constant from single-exponential fit of the currents (note: the values are close to those we have published in [5] but represent a different set of data). (*  =  p<0.05; **  =  p<0.01, unpaired t-test; n depicts the number of experiments)

**Figure 6:**
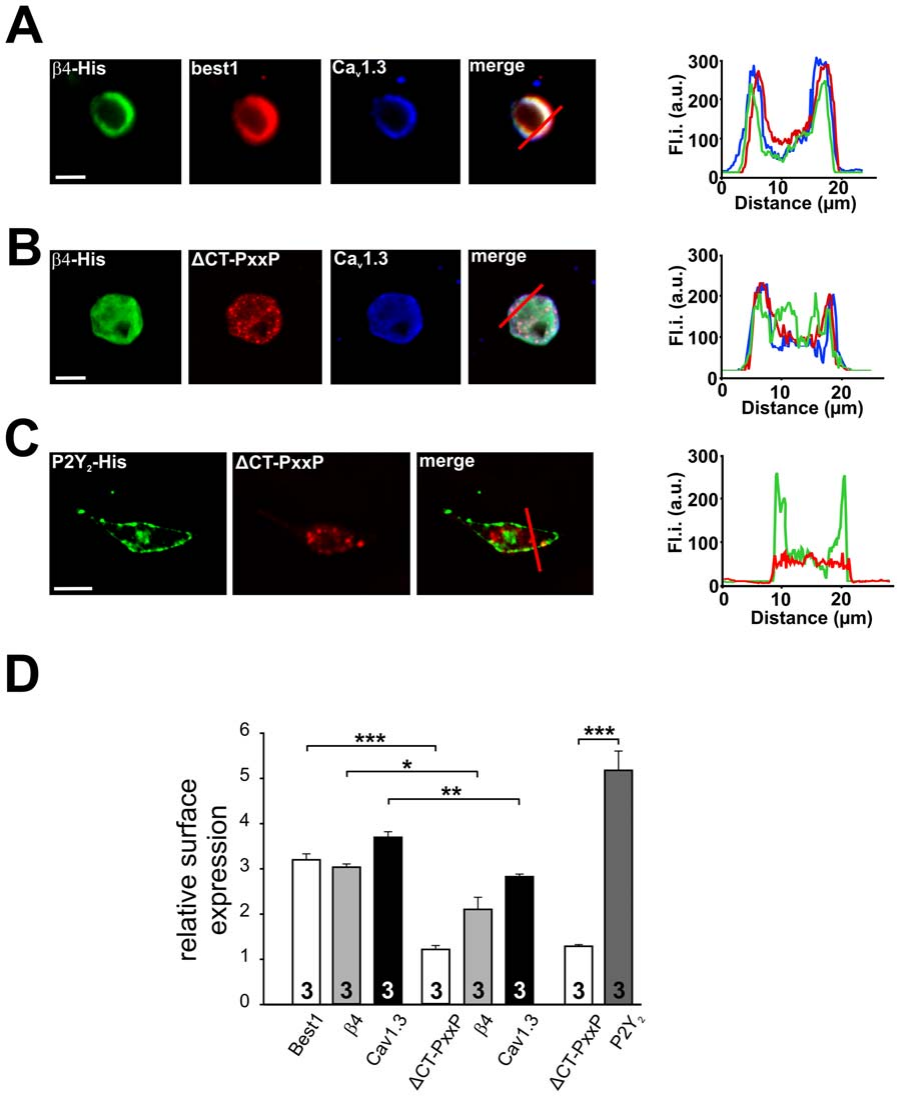
Subcellular localization of Ca^2+^ channel subunits and bestrophin-1 in cells used for patch-clamp analysis. **6A**: Confocal pictures of CHO cells expressing wt-bestrophin-1, β4-subunit, α2δ1-subunit and Ca_V_1.3. The merged picture and the fluorescence profiles measured along the red line (right panel) indicate the presence of β4-subunit, Ca_V_1.3 and wt-bestrophin-1 in the cell membrane. **6B**: Confocal pictures of CHO cells expressing ΔCTPxxP bestrophin-1, β4-subunit, α2δ1-subunit and Ca_V_1.3. The merged picture and the fluorescence profiles measured along the red line (right panel) indicate the presence of β4-subunit, Ca_V_1.3 and ΔCTPxxP bestrophin-1 in the cell plasma. **6C**: Confocal pictures of CHO cells expressing ΔCTPxxP bestrophin-1 and P2Y_2_ receptor. The merged picture and the fluorescence profiles measured along the red line (right panel) indicate the presence of ΔCTPxxP bestrophin-1 in the cell plasma whereas the P2Y receptor appears in the cell membrane. **6D**: Relative surface expression quantified by edge detection analysis (data are mean ± SEM; n = 3). (*  =  p<0.05; **  =  p<0.01, unpaired t-test) Scale bar represents 10 µm.

To study the functional implications of the β-subunit and bestrophin-1 interaction, patch-clamp analysis of Ca^2+^ channel currents of heterologously expressed Ca_V_1.3/β4 channels was performed (Figure 5). We chose β4-subunits because they are known to be expressed in the RPE. To obtain stable membrane localization and reliable membrane current measurements, α2δ1-subunits were additionally expressed. CHO cells were microinjected with all required plasmids. Cells which expressed Ca_V_1.3, β4-subunit and α2δ1 subunits showed Ba^2+^ currents associated with L-type channels as previously reported (Figure 5A; see Table 2) [5], [28], [29], [30]. To analyze whether the change in Ba^2+^ current density is due to modulation of the Ca_V_1.3 pore or due to less Ca_V_1.3 protein in the cell membrane, gating currents of Ca_V_1.3 channels were measured. When ionic currents were blocked after removal of Ba^2+^ and addition of Co^2+^, depolarization led to voltage dependent outward currents which activated at membrane potentials more positive than −32.45±2.0 mV (n = 5; Figure 5B/C). These currents represent the gating currents of the Ca_V_1.3 subunit [27]. The maximal ionic Ba^2+^ current density was reduced from 15.63±2.19 pApF^−1^ (n = 10) to 8.95±1.40 pApF^−1^ (n = 11; p = 0.0169 unpaired t-test) by the additional presence of wild-type bestrophin-1 (Figure 5D). Without bestrophin-1 the gating current density of 1.33±0.122 pApF^−1^ (n = 5; Figure 5E) was not significantly different compared to the gating current density in the presence of bestrophin-1 (1.24±0.26 pApF^−1^; n = 4; p = 0.75 unpaired t-test). However, in the presence of the ΔCTPxxP bestrophin-1 mutant the current density was with values of 5.03±0.44 pApF^−1^ (n = 8) further decreased compared to that in the presence wild-type bestrophin-1 (Figure 5D; p = 0.034 unpaired t-test). In the presence of ΔCT-PxxP, the gating currents were reduced to a density of 0.13±0.033 pApF^−1^ (n = 4; p = 0.0001 unpaired t-test; Figure 5E) indicating less Ca_V_1.3 protein in the cell membrane. The voltage-dependent activation of the currents was analyzed by fitting the normalized current/voltage relation by the Boltzmann function. Neither the voltage of half maximal activation nor the slope is significantly changed in the presence of bestrophin-1 (Figure 5F/G). In the presence of wild-type bestrophin-1, we found an acceleration of the time-dependent activation (Figure 5H) with an activation time constant at +20 mV of 1.26±0.15 ms (n = 11) versus 2.30±0.19 ms (n = 10; p = 0.0005 unpaired t-test) under control conditions without bestrophin-1 (Figure 5I). Using the ΔCT-PXXP form of the bestrophin-1 the Ca_V_1.3/β4 currents showed acceleration of time-dependent activation (activation time constant of 1.18±0.18 ms; n = 8) comparable to that in the presence of wild-type bestrophin-1 (Figure 5H/I). No differences of voltage-dependence of currents in the presence of the ΔCT-PxxP mutant compared to that in the presence of wild-type bestrophin-1 were observed (Figure 5F/G). CHO cells were analyzed by confocal microscopy after patch-clamp analysis (Figure 6). Ca_V_1.3 and β4-subunits were localized in the cell membrane which was not changed by the presence of wild-type bestrophin-1. Under these conditions all three proteins were found in the cell membrane (Figure 6A, Table 1) with relative surface expression values of 3.19±0.13 for bestrophin-1, 3.02±0.07 for β4-subunits and 3.67±0.122 for Ca_V_1.3 (all n = 3; Figure 6D). However, in the presence of ΔCT-PxxP mutant, a larger proportion of bestrophin-1, Ca_V_1.3 and β4-subunit were found in the cytosol (Figure 6B) indicated by relative surface expression values of 1.2±0.085 for ΔCTPxxP bestrophin−1, 2.1±0.266 for β4-subunits and 2.8±0.057 for Ca_V_1.3 subunits (all n = 3; Figure 6D). The surface expression values measured in the presence of wild-type bestrophin-1 were significantly different to those measured in the presence of ΔCTPxxP bestrophin-1 (p = 0.02–0.002). As a control comparison, the effect of ΔCTPxxP mutant on non-interacting protein P2Y_2_ receptor [31] was investigated by the same means (Figure 6C). Here the P2Y_2_ receptor was found in the cell membrane (surface expression value 5.17±0.42; n = 3) whereas the ΔCTPxxP bestrophin-1 was found in the cytoplasm (surface expression value 1.29±0.03; n = 3) indicating an independent trafficking of the two proteins (p = 0.0008).

**Table 2:** Summary of basic electrophysiological data measured at +20 mV (except voltage of maximal current amplitude) of Ca_V_1.3 channel currents in the presence of different β-subunits and bestrophins.

Transfection	Current density (pApF^−1^)	Activation time constant (ms)	V_1/2_ (mV)	V _max_ (mV)	n
β4-subunit control	15.63±2.19	2.30±0.19	−24.59±0.73	−7.00±2.13	10
β4-subunit + bestrophin-1	8.95±1.4	1.26±0.15	−23.44±0.91	−2.86±1.94	11
β4-subunit + ΔCTPxxP-best	5.03±0.44	1.18±0.18	−22.73±1.5	−2.50±2.5	8

P-values of statistical differences are indicated in the figures and in the text. (note: the values are close to those we have [5] but represent a different set of data).

## Discussion

In four independent studies bestrophin-1 appears to function as a regulator of voltage-dependent L-type Ca^2+^ channels [2], [3], [4], [5]. We have newly discovered an additional cluster of highly conserved proline-rich motifs on the C-terminus of bestrophin-1 and show that this cluster is required for bestrophin-1 -dependent modulation of β-subunit function.

In order to study direct interaction of bestrophin-1 with Ca^2+^ channel subunits, co-immunoprecipitation and co-localization experiments of heterologously expressed bestrophin-1 and different Ca^2+^ channel subunits were performed. In our system, co-precipitation of Ca_V_1.3 subunits with its physiological interaction partner β3-subunits could be observed [6], [7]. Co-precipitation was independent of the expression system. Co-localization detection and co-precipitation were dependent on certain amino acid motifs on the C-terminus of bestrophin-1. Thus our experimental system allowed detecting physiological interaction between Ca^2+^ channel subunits and regulatory proteins.

Heterologously expressed bestrophin-1 showed co-precipitation with β3- or β4-subunits but not with Ca_V_1.3 subunits. In the presence of β-subunits precipitation of Ca_V_1.3 subunits resulted in indirect co-precipitation of bestrophin-1. Thus Ca_V_1.3/β-subunits can form complexes with bestrophin-1 via binding of bestrophin-1 with β-subunits. Confocal microscopy of cells transfected with bestrophin-1 and β3-subunits showed a co-localization of the two proteins which was however more uniformly distributed in the cytoplasm. When the cells were transfected with Ca_V_1.3, β3-subunit and bestrophin-1 or Ca_V_1.3, β4-subunit and bestrophin-1, all three proteins were found to be localized in the cell membrane. This indicates close and direct interaction of bestrophin-1 with Ca^2+^ channel β-subunits. However, the methods used here could only indicate direct interaction. A stronger proof of this interaction would require experiments showing detection of FRET (fluorescence resonance energy transfer) which is beyond the scope of this study. The presence of wild-type bestrophin-1 had two effects on the Ca_V_1.3/β4 currents: an acceleration of the time-dependent activation and a reduction of ionic current density. The acceleration of the time-dependent activation has also been previously reported for β2-subunit modulation of Ca_V_1.2 currents in heterologous expression and endogenously expressed L-type channels in a RPE cell line [2], [3]. The reduction in the maximal activity was reported for β1-, β2- and β4-subunit/bestrophin-1 interaction in the modulation of rat Ca_V_1.3 currents [4] and for human Ca_V_1.3/β4-subunit currents [5]. Since the gating currents were not different in the absence or presence of bestrophin-1, the reduction of the ionic current density was most likely not due to a reduced number of Ca_V_1.3 subunits in the cell membrane. Thus, wild-type bestrophin-1 influences the ability of β-subunits to modulate the pore-function of Ca_V_1.3 subunits. This differs from observations made by Yu et al. [4] who used only the C-terminus of bestrophin-1 and not full length bestrophin-1 for gating current analysis.

The binding of β-subunits and bestrophin-1 could depend on the interaction between SH3 domains of β-subunits [6], [7], [32] with proline-rich motifs, PxxP, present on the C-terminus of bestrophin-1. One cluster with two PxxP motifs is between the amino acid positions 330 and 346 and has been reported to be responsible for bestrophin-1/β-subunit interaction [4]. We found another cluster located between the amino acid positions 468-486 containing four PxxP motifs. To study its functional role, we made a deletion mutant lacking the PxxP motifs between amino acid positions 468-486 (named ΔCTPxxP). This mutant showed a reduced efficiency to co-precipitate with β-subunits by 70-80% depending on the isoform of β-subunit. However, the weak co-precipitation of ΔCTPxxP bestrophin-1 with β-subunits might result from the PxxP motifs between amino acid positions 330-346 which are still present. Furthermore, when studying indirect co-precipiation of Ca_V_1.3/β4-subunit complex with bestrophin-1, we found no difference between wild-type bestrophin-1 and ΔCTPxxP mutant bestrophin-1. This can be explained by the occlusion of the SH3 domains in the free β-subunit crystal structure. [33], [34], [35]. It is hypothesized that the SH3 becomes accessible when the β-subunits bind to the Ca_V_-subunits [36]. Thus, bestrophin-1 can probably bind to β-subunits with higher efficiency when β-subunits are part of the Ca_V_1.3/β-subunit complex.

The functional effect of PxxP motifs deletion between the amino acid positions 468-486 was studied by patch-clamp analysis of currents through human Ca_V_1.3 subunit/β4-subunits expressed together with ΔCTPxxP-bestrophin-1. The presence of the ΔCTPxxP mutant only further decreased the ionic current density. Analysis of the subcellular localization revealed that these cells have a larger proportion of ΔCTPxxP bestrophin-1 and Ca_V_1.3 subunits in the cytoplasm. Furthermore, in the presence of ΔCTPxxP bestrophin-1, the gating current density was strongly reduced compared to that of wild-type bestrophin-1. Both observations indicate a reduced number of pore-forming Ca_V_1.3 subunits in the cell membrane in the presence of ΔCTPxxP bestrophin-1. Since the ΔCTPxxP mutant bestrophin-1 still binds to the Ca_V_1.3/β4-subunit complex, the reduced number of Ca_V_1.3 subunits is due to an influence of the ΔCTPxxP bestrophin-1 on the ability of β-subunits to regulate Ca_V_-subunit surface expression. Thus, when the PxxP motifs between amino acid positions 468–486 on the bestrophin-1 C-terminus are lacking β-subunits show reduced ability to enhance surface expression of Ca_V_1.3 subunits. To show that the effect of the ΔCTPxxP bestrophin-1 specifically influences trafficking Ca^2+^ channel proteins, we expressed the ΔCTPxxP bestrophin-1 together with the P2Y_2_ receptor which is known not to interact with bestrophin-1 [31]. Here, the P2Y receptor trafficked into the cell membrane whereas ΔCTPxxP bestrophin-1 stayed in the cytoplasm. Thus, the reduced amount of Ca^2+^ channel protein in the cell membrane by the presence by ΔCTPxxP bestrophin-1 is not due to unspecific protein aggregation in the cytosol. In addition, it should be noted that the ionic current density was reduced by 40% but the gating current density was reduced tenfold. Thus, the ΔCTPxxP mutant lost its ability to decrease the single channel conductance compared to wild-type bestrophin-1. In summary, the loss of the PxxP cluster between the amino acid positions 468–486 did not alter the binding of bestrophin-1 to the Ca_V_1.3/β-subunit complex but reduced the ability of β-subunits to guide pore-forming Ca_V_-subunits to the cell membrane and possibly influences the gating behavior of the Ca_V_1.3 subunit.

β-subunits modulate electrophysiological properties of the pore-forming Ca_V_-subunits, help to transport Ca_V_-subunits into the cell membrane and interact with protein kinases for further modulation of Ca^2+^ channel activity [8], [9]. The absence of the PxxP motifs between amino acid positions 468–486 of bestrophin-1 impaired the ability of β-subunits to regulate the surface expression of pore-forming Ca_V_-subunits and possibly modulate single channel properties of the Ca_V_1.3 channel. The absence of the PxxP motifs between the amino acid positions 330–346 reduced binding to β-subunits but did not affect the trafficking of Ca_V_1.3 subunits to the cell membrane [5]. Thus, the PxxP motifs between amino acid positions 330–346 are more responsible for the binding of β-subunits and bestrophin-1 and the PxxP motifs between amino acid positions 468-486 help maintain the β-subunit regulatory properties over the surface expression of Ca_V_-subunits.

We demonstrated that bestrophin-1 modulates human Ca_V_1.3/β4-subunits which are expressed in the RPE. Morbus Best patients show reduced light-peak in the electro-oculogram [13], [37], [38]. Mouse models which show the same phenotype as Best patients are the Ca_V_1.3 knock-out mouse [17] and the lethargic mice which are a natural knock-out of the β4-subunit [16]. Thus Ca_V_1.3/β4-subunits are of importance in the generation of the light-peak. The decreased light-peak in Best patients would probably result from altered modulation of L-type channels composed of Ca_V_1.3/β4 subunits. Since L-type channels of the RPE regulate cell functions such as secretion or phagocytosis, the understanding of bestrophin-1 and Ca^2+^ channel interaction would then help to understand mechanisms of retinal degeneration [39], [40].

In summary, we were able to demonstrate the physical binding of full length bestrophin-1 with β3- and β4-subunits of Ca^2+^ channels. Additionally, we identified a new cluster of PxxP motifs on the C-terminus of bestrophin-1 which is needed for the interaction of bestrophin-1 with Ca^2+^ channel β-subunits and probably other SH3 domain carrying proteins.

## Supporting Information

Figure S1 **S1A: Control experiments**: Proteins detected in transfected CHO cells are products of the plasmids used for transfection. Western blot of proteins isolated from CHO cells either transfected or not transfected by the corresponding plasmid. Only when the cells were transfected by plasmids carrying bestrophin-1, β3-subunit, bestrophin-1-GFP or Ca_V_1.3 subunits the Western blots revealed the presence of the corresponding proteins (bestropin-1; 68kDa, β3 subunit; 55kDa, bestrophin-1-GFP; 100kDa, and Cav1.3 subunit; 240 kDa). Anti β-actin antibody was used as a loading control. **S1B**: Physical interaction between bestrophin-1 and auxiliary β3-subunits of voltage-dependent Ca^2+^ channels was independent from the cell line which was used as transfection system: CHO, COS-7, and HEK-293 cells were co-transfected with bestrophin-1-GFP and β3-subunits, and β3-subunits and GFP. Precipitates were obtained using anti GFP antibodies, and Western blots were stained using anti-β3 and anti-GFP antibodies. β3-subunits were detected only when cells were transfected with bestrophin-1-GFP fusion construct and β3-subunits but not when cells were transfected with β3-subunits and GFP vector.
(TIF)

Figure S2 **S2A**: Localization of β3-subunit in the cell membrane shown by detection after application of edge detection tool on fluorescence signals. **S2B**: Left panel: Calculation of surface expression ratios. Left panel showing the first step: measurement of the pixel number inside the area surrounding the cell. Right panel showing the second step: measurement of the pixel number inside the area encirculating the cytoplasm. **S2C**: Example of proteins distributed in the cytoplasm shown after edge detection. (bar represents 20 µm)
(TIF)

Figure S3 **S3A**: Comparison of detection and immunoprecipitation efficiency of wild-type bestrophin-1 and ΔCTPxxP bestrophin-1. **S3B**: Comparison of detection and immunoprecipitation efficiency of His-tagged β4-subunits expressed together with either wild-type bestrophin-1 or ΔCTPxxP bestrophin-1. (L  =  lysate; IP  =  immunoprecipitation; NB  =  not bound)
(TIF)
