# Supplementary figures and images for: Interaction of Bestrophin-1 and Ca2+ Channel β-Subunits: Identification of New Binding Domains on the Bestrophin-1 C-Terminus
Source: PLoS One. 2011 Apr 29;6(4):e19364. doi: 10.1371/journal.pone.0019364 (PMC3084833; doi:10.1371/journal.pone.0019364)

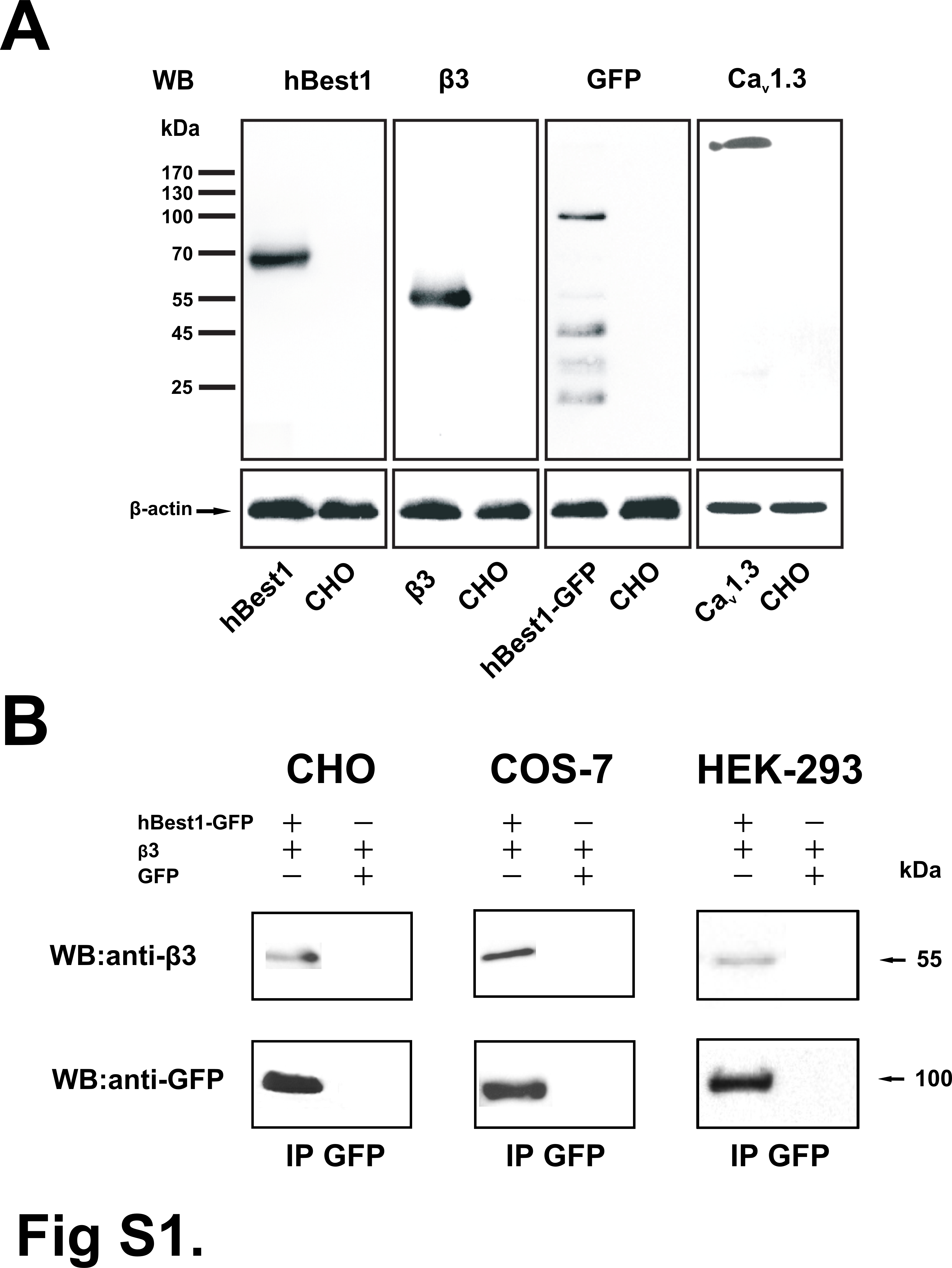

Supplement: Figure S1 — S1A: Control experiments: Proteins detected in transfected CHO cells are products of the plasmids used for transfection. Western blot of proteins isolated from CHO cells either transfected or not transfected by the corresponding plasmid. Only when the cells were transfected by plasmids carrying bestrophin-1, β3-subunit, bestrophin-1-GFP or CaV1.3 subunits the Western blots revealed the presence of the corresponding proteins (bestropin-1; 68kDa, β3 subunit; 55kDa, bestrophin-1-GFP; 100kDa, and Cav1.3 subunit; 240 kDa). Anti β-actin antibody was used as a loading control. S1B: Physical interaction between bestrophin-1 and auxiliary β3-subunits of voltage-dependent Ca2+ channels was independent from the cell line which was used as transfection system: CHO, COS-7, and HEK-293 cells were co-transfected with bestrophin-1-GFP and β3-subunits, and β3-subunits and GFP. Precipitates were obtained using anti GFP antibodies, and Western blots were stained using anti-β3 and anti-GFP antibodies. β3-subunits were detected only when cells were transfected with bestrophin-1-GFP fusion construct and β3-subunits but not when cells were transfected with β3-subunits and GFP vector. (TIF) [file pone.0019364.s001.tif]

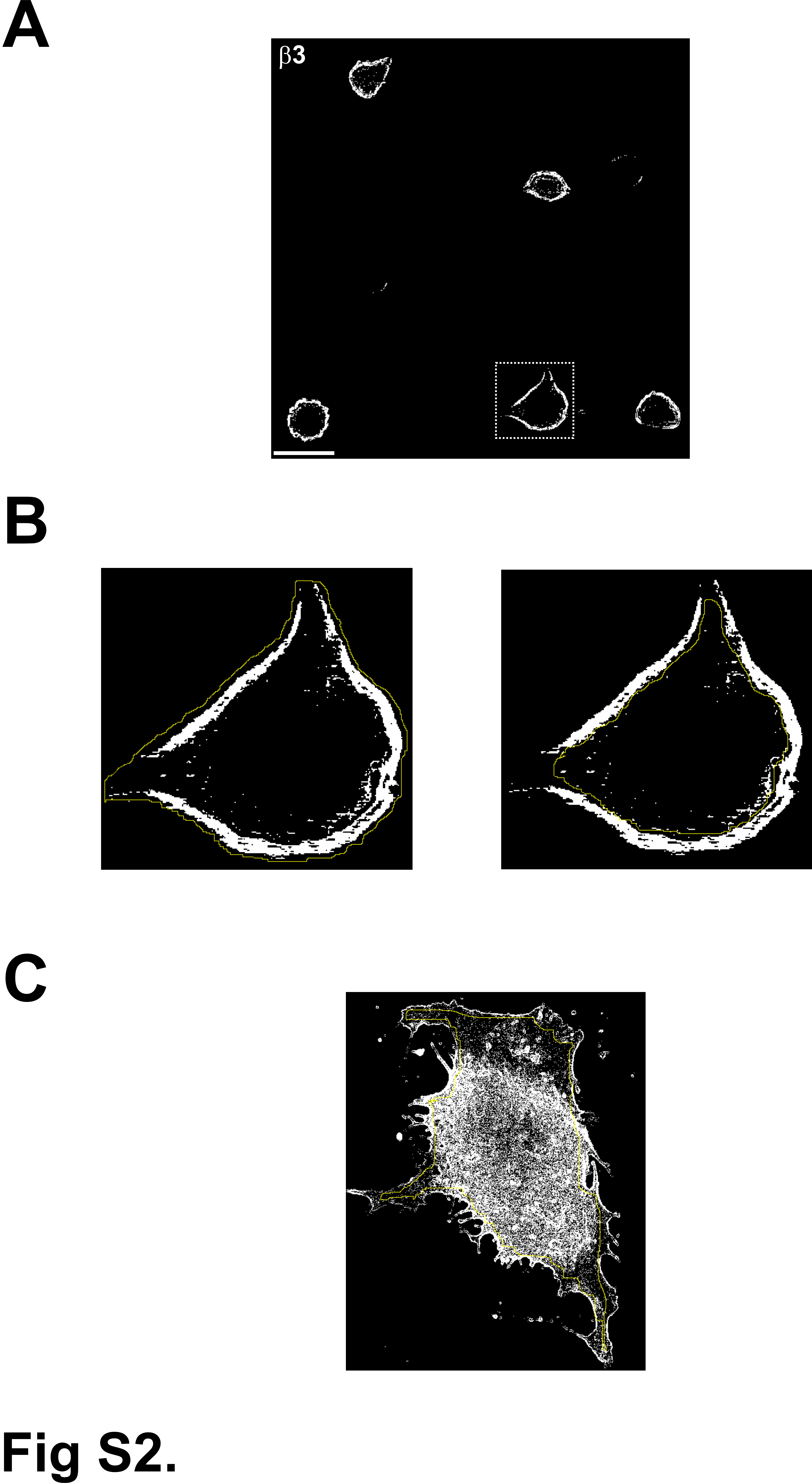

Supplement: Figure S2 — S2A: Localization of β3-subunit in the cell membrane shown by detection after application of edge detection tool on fluorescence signals. S2B: Left panel: Calculation of surface expression ratios. Left panel showing the first step: measurement of the pixel number inside the area surrounding the cell. Right panel showing the second step: measurement of the pixel number inside the area encirculating the cytoplasm. S2C: Example of proteins distributed in the cytoplasm shown after edge detection. (bar represents 20 µm) (TIF) [file pone.0019364.s002.tif]

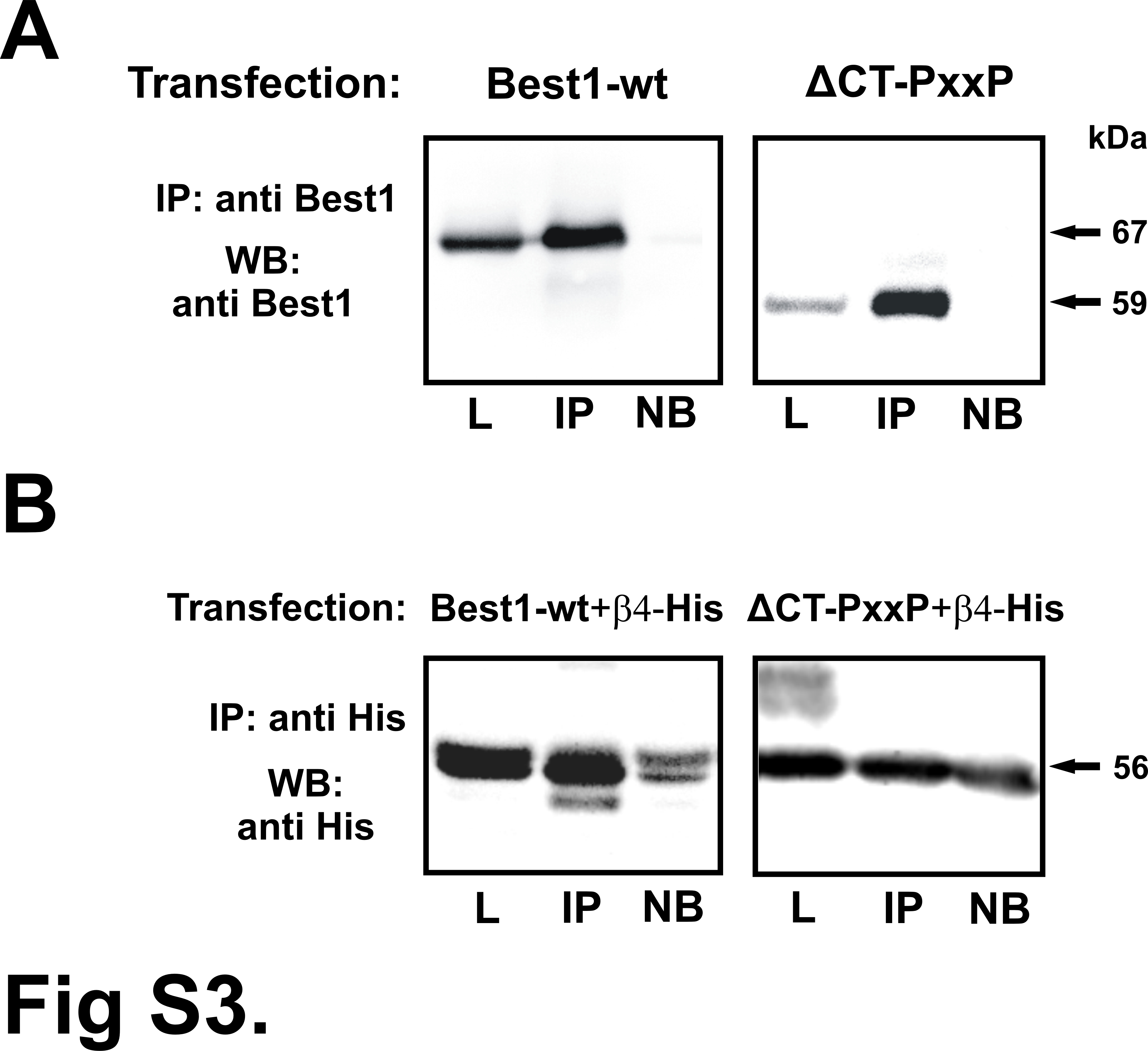

Supplement: Figure S3 — S3A: Comparison of detection and immunoprecipitation efficiency of wild-type bestrophin-1 and ΔCTPxxP bestrophin-1. S3B: Comparison of detection and immunoprecipitation efficiency of His-tagged β4-subunits expressed together with either wild-type bestrophin-1 or ΔCTPxxP bestrophin-1. (L = lysate; IP = immunoprecipitation; NB = not bound) (TIF) [file pone.0019364.s003.tif]
